# Editorial: Plant architecture: structure, development, evolution, and molecular/genetic regulation

**DOI:** 10.3389/fpls.2023.1211224

**Published:** 2023-08-16

**Authors:** Abelardo Carlos Vegetti

**Affiliations:** Instituto de Ciencias Agropecuarias del Litoral, Consejo Nacional de Investigaciones Científicas y Técnicas (CONICET) – Universidad Nacional del Litoral, Esperanza, Santa Fe, Argentina

**Keywords:** plant architecture, development, phylogeny, genetic regulation, environmental stresses

Plant architecture has important ecological and agronomic management implications and also aids in establishing taxonomic and phylogenetic relationships. It is of major agronomic importance since plant architecture is highly modified during the domestication of crops. Such structural changes in the plant body have greatly modified the behavior of crops in different environments, including stressful ones, such as their yield and harvesting efficiency.

This Research Topic covers the following subtopics:

1. Plant structural patterns;
2. Molecular-genetic regulation of these structural patterns;
3. Environmental conditions/abiotic stresses and modifications of these structural patterns;
4. Evolution of plant architecture/plant form/structural patterns.

The investigations within this Research Topic analyze issues related to the architecture of the plant, the architecture of the inflorescence, and its strong and intricate relationship with the performance components. Aspects related to metabolic and molecular issues that regulate the adaptation of plants to biotic and abiotic stress and the importance of these issues in relation to climate change are explored. From the histological point of view, the genetic and molecular bases of the development of the periderm and the vascular cambium and their productions are studied.

The inflorescence is one of the main organs for determining grain yield. The genetic and molecular regulation of rice inflorescence architecture has been well investigated in recent years. The study by Chun et al. describes the genes that regulate rice inflorescence architecture based on their roles in maintaining meristem activity, meristem identity conversion, and branch elongation. The emerging regulatory pathways of phytohormones involved in rice inflorescence development are also presented. This study shows the complexities and challenges of manipulating inflorescence architecture to improve rice yield.

Plant developmental dynamics not only affect ecological adaptation but also contribute to the realization of genetically determined yield potentials in various environments. For wheat, the combination of insensitivity and sensitivity to the photoperiod response gene (PPD-D1) with the extremes of the early or late alleles at the corresponding minor developmental loci resulted in distinct and significantly altered plant development patterns with detectable results in some performance-related traits. The dissection of the genetic determinants of plant development is urgent due to global climate change, which can severely affect and even alter locally adapted development patterns (Horváth et al.).

Nudix hydrolases (NUDX) are widely involved in biotic and abiotic stress responses in different plant species; however, their role in plant growth and development remains largely unknown. In the investigation by Liu et al., OsNUDX14 located in rice mitochondria is identified and characterized. The results showed that OsNUDX14 is constitutively expressed in various tissues and is more strongly expressed in mature leaves. OsNUDX14 is involved in plant development and grain quality regulation, providing a potential opportunity to optimize plant architecture and quality for crop improvement.

A review by Wang et al. on the development of the periderm was carried out, proposing the conservation of the regulation of the periderm between fruits and other organs of the plant based on their morphological and molecular characteristics, and synthesizing a regulatory network with elicitors and repressors for development. of tissues. Programmed spontaneous cell death (PCD) or environmental stress produces the original signal that triggers periderm development. The spatio-temporal specific PCD produced by the PyPPCD1 gene and its homologs may play a key role in the coordinated regulation of cell death-related tissue development.

Multiple hormonal signals and endogenous developmental programs are involved in the regulation of vascular cambium activity. Brassinosteroids (BR) significantly promote secondary stem growth and wood formation in poplars. However, the underlying regulatory mechanisms of BRs within vascular tissue remain largely unknown. In an investigation by Wang et al. within this Research Topic, genetic and anatomical approaches are used to elucidate the role of PagDET2, the rate-limiting enzyme for BR biosynthesis, in regulating the activity of the secondary vascular cambium in Populus. RNA-seq analysis revealed that genes involved in BR responses and vascular cambium cell division, differentiation, synthesis, and regulation are upregulated in response to increased BR levels of PagDET2 overexpression lines. This study characterizes the changes in vascular cambium cell activity in response to elevated BR and outlines the candidate BR-responsive genes that function in vascular cambium cell division and xylem differentiation in poplars.

The six articles included in this Research Topic contribute to the theme of *Plant Architecture: Structure, Development, Evolution, and Molecular/Genetic Regulation*, which is part of the Research Topic. This editorial introduces a collection of articles on the Research Topic of how changes in plant architecture allow plants to adapt to different environments and yield more agricultural products.

A better understanding of the structural patterns and the molecular-genetic regulation of the plant form will enable further knowledge of the development and morphogenesis processes of plants, providing relevant information for crop improvement.

This comprehensive Research Topic covers a variety of plant structures, as well as several economically important crop species. It is very interesting to focus on the genetic basis for plant architecture as the intersection between environment and plant morphology. Under the current global climate crisis, the publication of this Research Topic is very timely.

Despite some progress in this field, the understanding of the morphogenetic processes and the development of plant architecture remains limited. Therefore, new investigations are required that cover architectural, histological, developmental, genetic, and molecular aspects and their relationship with environmental factors (abiotic stress).

## Author contributions

The author confirms being the sole contributor of this work and has approved it for publication.

